# Metabolic and inflammatory mechanisms in uric acid-induced tubular dysfunction: Emerging perspectives

**DOI:** 10.1016/j.clinsp.2026.100958

**Published:** 2026-04-22

**Authors:** Caixiu Yang, Shuo Xu, Hongmei Zhao, Yanwen Wang, Dongmei Meng, Qiuxia Ji

**Affiliations:** a Department of Periodontology, The Affiliated Hospital of Qingdao University, Qingdao, China; b School of Stomatology of Qingdao University, Qingdao, China; c Department of Periodontology, Shanghai Ninth People's Hospital, Shanghai Jiao Tong University School of Medicine, Shanghai, China; d Department of Prosthodontics, The Affiliated Hospital of Qingdao University, Qingdao, China; e Department of Biological Sciences, Carnegie Mellon University, Pittsburgh, PA 15213, USA; f Department of Endocrinology and Metabolic Disorders, The Affiliated Hospital of Qingdao University, Qingdao, China

Hyperuricemia is a common metabolic disorder and is the direct cause of gout and Uric Acid (UA) nephrolithiasis.^1^, ^2^, ^3^, ^4^ Increasing evidence indicates that elevated serum UA is not only a consequence of kidney disease but also a critical risk factor contributing to renal dysfunction.^5^^,^^6^ The kidney, particularly the proximal tubule, plays a pivotal role in urate homeostasis through a coordinated network of transporters, including reabsorptive proteins (URAT1, GLUT9) and secretory transporters (*ABCG2*, OAT3).^7^, ^8^, ^9^, ^10^ Dysregulation of this “reabsorption-excretion” balance can lead to urate accumulation and downstream injury. However, the mechanisms by which UA itself perturbs tubular function and transporter expression has not been fully elucidated.

To investigate the effects of UA on renal tubular cells, human proximal tubular epithelial cells (HK-2) were exposed to increasing concentrations of UA (0–300 mg/dL) for 24 h, and cell viability was assessed via the CCK-8 assay. A concentration of 10 mg/dL, which approximates hyperuricemic levels without inducing overt cytotoxicity (Fig. 1A), was selected for mechanistic studies (Fig. 1A). qRT-PCR revealed that UA exposure downregulated excretory transporters (*ABCG2, SLC22A8*) while upregulating reabsorptive transporters (*SLC22A12, SLC2A9*), functionally shifting the balance toward increased reabsorption and decreased excretion (Fig. 1B). Transcriptomic profiling of HK-2 cells treated with 10 mg/dL UA for 24 h revealed significant enrichment in solute transport processes, particularly monovalent cation and amino acid transport (Fig. 1C). KEGG pathway analysis indicated activation of inflammatory signaling pathways ‒ including NOD-like receptor, cytokine-cytokine receptor interaction, and JAK-STAT cascades ‒ consistent with prior reports of UA-induced inflammation and mitochondrial stress.^11^, ^12^, ^13^ Enrichment of alanine, aspartate, and glutamate metabolism, together with oxidative phosphorylation and ROS-related pathways (Fig. 1D,E), suggested that metabolic reprogramming and oxidative stress might serve as central mediators linking UA to altered transporter expression. These findings indicate that exogenous UA can alter the transcriptional profile of HK2 cells, potentially creating a maladaptive feedback process characterized by impaired UA excretion and increased reabsorption. It is important to emphasize, however, that these results are derived from in vitro models, which may not fully recapitulate the complex homeostatic mechanisms in vivo. In intact organisms, compensatory regulatory pathways ‒ such as changes in renal hemodynamics, systemic oxidative balance, and hormonal control of urate handling ‒ may modulate or attenuate this proposed feedback.^14^, ^15^, ^16^ In addition, there is contradictory evidence indicating that UA may exert neutral or even protective effects on renal tubular under certain conditions.^17^^,^^18^

**Fig. 1:**
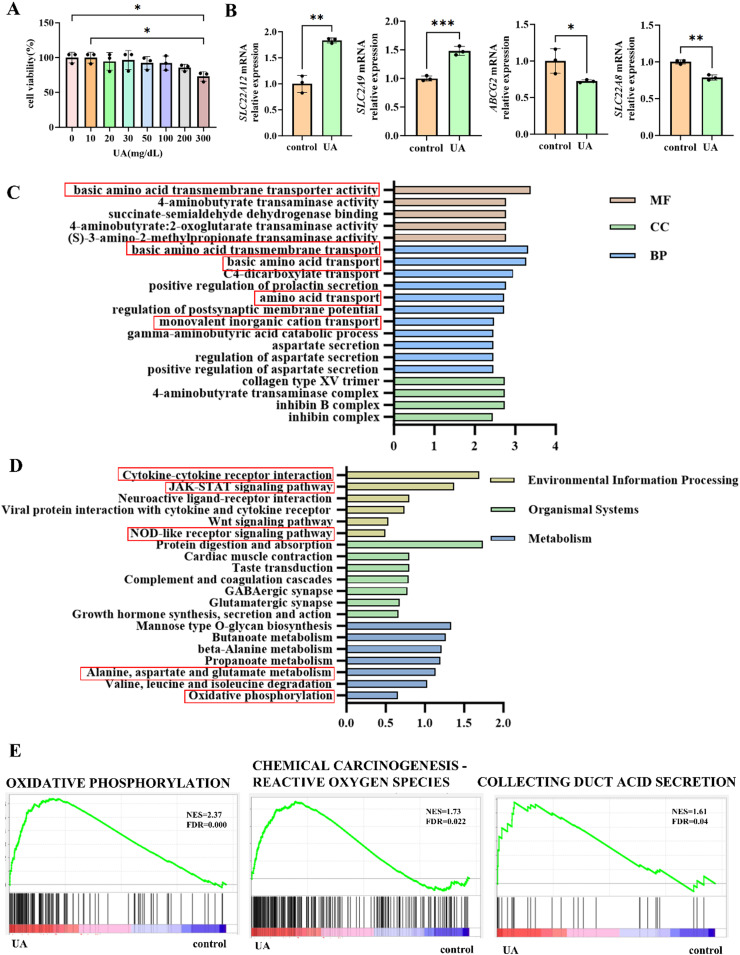
UA modulates the expression of genes related to UA excretion and reabsorption in HK2 cells through inflammatory signaling pathways. Groups: control and UA. (A) Cytotoxicity was assessed using the CCK-8 assay in HK2 cells treated with UA for 24 h. (B) Following 24 h of UA treatment, the expression of UA excretion and reabsorption-related genes in HK2 cells was quantified by qRT-PCR. (C) GO enrichment analysis revealed key biological processes altered in HK2 cells upon UA treatment. The region highlighted in red indicates significant changes in solute transport processes. (D) KEGG pathway analysis reveal significant enrichment of biological pathways in the UA-treated HK2 cell model. The magnified region highlighted in red specifically illustrates key inflammatory and metabolic pathways. (E) GSEA pathway analysis revealed significant enrichment of oxidative phosphorylation and ROS-related signaling pathways in the UA-treated HK2 cell model. (All experiments independently repeated ≥ 3-times; * p < 0.05, ** p < 0.01, *** p < 0.001, **** p < 0.0001).

Mechanistically, our data, together with previous studies, support a model in which UA may trigger mitochondrial oxidative stress, leading to activation of the NLRP3 inflammasome and JAK-STAT signaling.^19^^,^^20^ These cascades converge on transcription factors such as NF-κB and STAT3, which may regulate transporter gene expression, leading to reduced excretory capacity (*ABCG2*, OAT3) and enhanced reabsorption (URAT1, GLUT9).^21^^,^^22^ Nevertheless, this interpretation should be viewed as a hypothesis that integrates existing molecular and physiological evidence rather than a definitive causal pathway. Future investigations using in vivo systems and genetic models will be essential to delineate whether such a maladaptive feedback process truly operates within the physiological context. Additionally, the regulation of transporters by UA may involve complex post-transcriptional processes beyond transcriptional control. Previous studies have demonstrated that UA can modulate transporter function through multiple mechanisms, including altering subcellular localization and influencing post-translational modifications.^23^^,^^24^ Notably, the effects of UA may not be restricted to the proximal tubule; enrichment of acid-secretion pathways in the collecting duct suggests that distal nephron function may also be affected. Taken together, these observations delineate a molecular framework in which UA modulates transporter expression through the oxidative stress-inflammation axis, providing new insights into tubular transport function and injury.

Collectively, UA appears to reprogram renal tubular function through intersecting metabolic and inflammatory pathways. The ROS-inflammation axis likely represents a crucial regulatory hub contributing to urate retention and tubulointerstitial injury. Recognizing this complexity highlights not only potential therapeutic targets but also the need for integrated approaches that account for the multifactorial nature of uric acid homeostasis.

## Authors’ contributions

Caixiu Yang: Conceptualization; Methodology; Formal analysis; Writing-original draft.

Hongmei Zhao: Data curation; Writing-review and editing.

Dongmei Meng: Validation; Writing-review and editing.

Yanwen Wang: Supervision; Visualization.

Shuo Xu: Supervision.

Qiuxia Ji: Supervision; Project administration; Funding acquisition.

## Data availability

The datasets generated and/or analyzed during the current study are available from the corresponding author upon reasonable request.

## Declaration of competing interest

The authors declare that they have no known competing financial interests or personal relationships that could have appeared to influence the work reported in this paper.

## References

[1] Chaisawat T., Wongprasopchai P., Kaopaiboon S. (2025). Nanoencapsulation of baccaurea macrophylla pericarp extract in nanostarch-based for in vitro xanthine oxidase inhibition. Int J Biol Macromol.

[2] Kaushal K., Goutam N., Sharma A., Shivani (2025). Phytotherapeutic insights into hyperuricemia: a mechanistic and clinical perspective. Inflammopharmacology.

[3] Richette P., Bardin T. (2010). Gout. Lancet.

[4] Preitner F., Bonny O., Laverrière A. (2009). Glut9 is a major regulator of urate homeostasis and its genetic inactivation induces hyperuricosuria and urate nephropathy. Proc Natl Acad Sci U S A..

[5] Song Y., Li Q., Lu J.F. (2025). Dietary purines and health: metabolism, impact, and regulation. Trends Food Sci Technol.

[6] Kang D.H., Nakagawa T., Feng L.L. (2002). A role for uric acid in the progression of renal disease. J Am Soc Nephrol.

[7] Enomoto A., Kimura H., Chairoungdua A. (2002). Molecular identification of a renal urate-anion exchanger that regulates blood urate levels. Nature.

[8] Novikov A., Fu Y.L., Huang W. (2019). SGLT2 inhibition and renal urate excretion: role of luminal glucose, GLUT9, and URAT1. Am J Physiol Renal Physiol.

[9] Hoque K.M., Dixon E.E., Lewis R.M. (2020). The ABCG2 Q141K hyperuricemia and gout associated variant illuminates the physiology of human urate excretion. Nat Commun.

[10] Xu G., Bhatnagar V., Wen G., Hamilton B.A., Eraly S.A., Nigam S.K. (2005). Analyses of coding region polymorphisms in apical and basolateral human organic anion transporter (OAT) genes OAT1 (NKT), OAT2, OAT3, OAT4, URAT (RST) ‒ Rapid Communication. Kidney Int.

[11] Benko S., Philpott D.J., Girardin S.E. (2008). The microbial and danger signals that activate nod-like receptors. Cytokine.

[12] Crisan T.O., Cleophas M.C.P., Oosting M. (2016). Soluble uric acid primes TLR-induced proinflammatory cytokine production by human primary cells via inhibition of IL-1Ra. Ann Rheum Dis.

[13] Sellmayr M., Petzsche M.R.H., Ma Q.Y. (2020). Only hyperuricemia with crystalluria, but not asymptomatic hyperuricemia, drives progression of chronic kidney disease. J Am Soc Nephrol.

[14] Zhou X.Y., Matavelli L., Frohlich E.D. (2006). Uric acid: its relationship to renal hemodynamics and the renal renin-angiotensin system. Curr Hypertens Rep.

[15] Zinellu A., Mangoni A.A. (2023). A systematic review and meta-analysis of the association between Uric Acid and Allantoin and Rheumatoid arthritis. Antioxidants.

[16] Yener S., Cömlekci A., Yuksel F., Sevinc A., Ertilav S., Yesil S. (2012). Traditional and novel cardiovascular risk factors in non-functioning adrenal adenomas. Eur J Intern Med.

[17] Miake J., Hisatome I., Tomita K. (2023). Impact of hyper- and Hypo-uricemia on kidney function. Biomedicines.

[18] Srinivasan S., Kalaiselvi P., Sakthivel R., Pragasam V., Muthu V., Varalakshmi P. (2005). Uric acid: an abettor or protector in calcium oxalate urolithiasis? Biochemical study in stone formers. Clin Chim Acta.

[19] Wang T., Wang D.F., Ren L.K. (2025). Preparation, functional mechanism, and deep-learning application of food-derived xanthine oxidase-inhibitory peptides: a comprehensive review. Trends Food Sci Technol.

[20] Zhang Z.J., Shi X.Y., Wu T. (2025). Discovery of multi-target anti-gout agents from Eurycoma longifolia Jack through phenotypic screening and structural optimization. Nat Commun.

[21] Li Y.Q., Zhang L.Y., Wu D.R. (2025). Kynurenic acid alleviated hyperuricemia and its complications by inhibiting xanthine oxidase. Food Biosci.

[22] Tong R., Ding B.A., Chen S.Y. (2025). Antihyperuricemic, hepatoprotective and nephroprotective roles of Benincasae Exocarpium in hyperuricemia rats. J Ethnopharmacol.

[23] Zhu Y.Q., Zhang B., He Y. (2025). Molecular interplay between TXNIP and GLUT9 underlies uric acid transport dysregulation in vitro under hyperuricemic stress. Eur J Med Res.

[24] Mozner O., Bartos Z., Zámbó B., Homolya L., Hegedus T., Sarkadi B. (2019). Cellular processing of the ABCG2 transporter-potential effects on gout and drug metabolism. Cells.

